# Microwave-Treated Structural and Network Changes in Filled Industrial EPDM Waste: Effects of Particle Size and Exposure Time

**DOI:** 10.1021/acsomega.6c04739

**Published:** 2026-10-07

**Authors:** Begüm Uzunbayir, Bahadir Uyulgan, Orçun Saf

**Affiliations:** † Dokuz Eylul University, The Graduate School of Natural and Applied Sciences, Department of Metallurgical and Materials Engineering, Buca, 35390 Izmir, Türkiye; ‡ Dokuz Eylul University, Department of Metallurgical and Materials Engineering, Buca, 35390 Izmir, Türkiye; § Standard Profil Ege, R&D Department, 45030 Manisa, Türkiye

## Abstract

Ethylene–propylene–diene monomer (EPDM) compounds used in automotive sealing systems contain permanent cross-links and high amounts of fillers and process oils, which complicate their recycling. This study examined the effects of particle-size fraction (≤125 and ≥125 μm) and microwave exposure time (60–180 s at 600 W) on the structural response of a highly filled industrial EPDM waste. Apparent sol fraction and apparent cross-link density were used as comparative indicators, supported by ATR-FTIR, XPS, TGA, and SEM analyses. The fine fraction treated for 60 s showed the lowest apparent cross-link density (0.72 × 10^–4^ mol/cm^3^), corresponding to an apparent reduction of 17.11% relative to untreated ground EPDM. Longer exposure is increasingly favored by network-forming responses, particularly in the coarse fraction, demonstrating competition between network loosening and secondary cross-linking. XPS showed changes in sulfur-containing surface environments, while ATR-FTIR revealed no appreciable shifts in the principal EPDM bands. TGA and SEM showed clear differences between the particle-size fractions. In revulcanized compounds containing 10 phr recycled EPDM, tensile strength remained within 7.71–8.32 MPa, and none of the recycled formulations differed significantly from the reference C-EPDM. Microwave treatment had a significant overall effect on tensile strength (*p* = 0.014), but only the treated fine fraction differed from its untreated counterpart (*p* = 0.046); elongation at break did not differ significantly. Surface roughness and Shore A hardness remained broadly comparable. Overall, microwave exposure produced competing particle-size- and time-dependent network responses, indicating a narrow processing window for the controlled modification of highly filled industrial EPDM waste.

## Graphical abstract

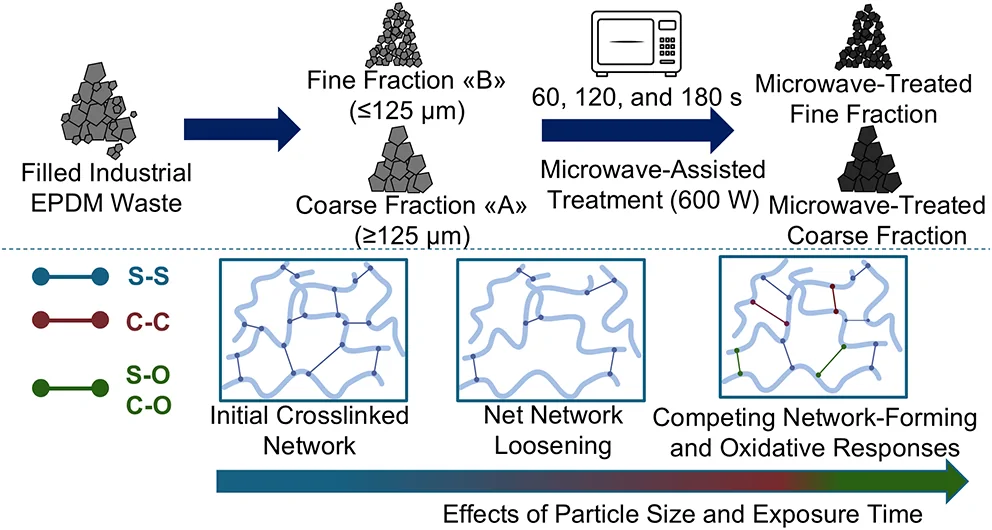

## Introduction

The transition toward a circular economy requires effective recycling strategies for commercial elastomers. Among them, EPDM (ethylene–propylene–diene monomer) is widely used in automotive sealing applications because of its resistance to heat, oxidation, ozone, and weathering.^1^ In these applications, EPDM is typically formulated as a highly filled compound containing carbon black, processing oils, antioxidants, and sulfur-based curing agents. Although vulcanization provides the cross-linked structure required for long-term mechanical and environmental performance, it also makes EPDM difficult to recycle by conventional remelting or remolding routes.^2^ As a result, rubber waste is often downgraded to low-value uses such as combustion or incorporation as untreated ground particles in new matrices.^3–7^ However, untreated rubber particles usually show poor compatibility with the surrounding matrix, which limits the performance of the final products. Therefore, improving the structural state and reprocessability of EPDM waste is essential for higher-value reuse.^3,8,9^

To address these limitations, devulcanization plays a key role in selective rubber recycling. This process functions as the reverse of vulcanization, selectively cleaving sulfur–sulfur (S–S) and carbon–sulfur (C–S) cross-links without degrading the carbon–carbon (C–C) bonds of the main polymer chain. The preservation of the polymer backbone is crucial, as it allows the material to regain the ability to be processed, reshaped, and reintegrated into its original applications.^10^ To this end, chemical, biological, and physical methods have been explored for rubber devulcanization.^11^ In practice, however, the response of industrial rubber compounds cannot be described as purely selective cross-link cleavage, because competing reactions may also occur during treatment. Achieving cross-link cleavage without inducing undesirable side reactions, therefore, remains a major challenge.^12–14^

Among the various devulcanization methods investigated, microwave-assisted treatment has attracted considerable attention as a promising approach.^15–17^ In 1976, microwave devulcanization was first applied to waste tire rubber (WTR) by The Goodyear Tire & Rubber Company.^18,19^ Microwave-assisted treatment is attractive because it provides rapid and volumetric heating through mechanisms such as dipole rotation, ionic conduction, and interfacial polarization. However, nonpolar rubbers such as EPDM do not efficiently absorb microwave irradiation and therefore cannot directly convert electromagnetic energy into heat.^20–22^ Therefore, microwave-induced heating in such systems is largely governed by the presence of carbon black, which absorbs microwave energy through interfacial polarization (Maxwell-Wagner effect) and transfers heat to the rubber matrix. Such filler-mediated heating may enhance network loosening but can also generate heterogeneous thermal fields and treatment-induced structural changes under severe conditions.^23^ This issue is particularly important in highly filled industrial EPDM compounds, where localized heating may promote sulfur-network cleavage, nonselective chain scission, oxidation, surface modification, and secondary cross-link formation. Accordingly, the response of these compounds may not be fully described within the conventional framework of selective devulcanization.

Numerous studies have reported on the microwave-assisted devulcanization of various elastomers, especially ground tire rubbers (GTR), natural rubber (NR), styrene butadiene rubber (SBR), and waste EPDM. Besides, the effectiveness of microwave-assisted devulcanization is determined by the interplay among microwave power, exposure time, particle size, and the material’s energy-absorption capacity; even within the same system, varying conditions can yield significantly different results. Previous studies on natural rubber and GTR by de Sousa and co-workers^24^ showed that prolonged microwave exposure increases sample temperature and enhances devulcanization through a reduction in cross-link density, whereas Faizal et al.^25^ reported that, in EPDM waste, cross-link breakdown improved only up to an optimum treatment duration of 5 min, beyond which the trend reversed. Scuracchio et al. investigated the devulcanization of ground tire rubber (GTR) under constant microwave power (700 W) while varying the irradiation time (1 min to 5 min). Their results demonstrated that increasing exposure time led to a rise in rubber temperature, accompanied by a reduction in gel content, which indicates an enhanced degree of devulcanization. However, it was emphasized that the maximum irradiation time should be limited to less than 4 min in order to avoid rubber carbonization.^21,26^ Particle size has also been identified as an important factor, as Vahdatbin et al. demonstrated that large particles (>1 cm) tend to burn under microwave irradiation because of localized overheating, while smaller particles (e.g., 1 mm) enable more efficient devulcanization owing to a more homogeneous temperature distribution.^14^ Similarly, Aoudia et al. observed that microwave energy promotes devulcanization only up to a saturation threshold (approximately 1.389 kJ/kg of energy), beyond which additional energy contributes little to process efficiency.^12^ In industrial compounds, however, the situation is more complex because the presence of carbon black, oils, inorganic fillers, and other additives strongly modifies the heating behavior under microwave irradiation. Since EPDM is a nonpolar elastomer with inherently poor microwave absorption, fillers—particularly carbon black—become essential for converting electromagnetic energy into heat, mainly through Maxwell–Wagner polarization, as demonstrated by de Sousa,^27^ and Hirayama and co-workers.^28^ Zanchet et al. concluded that microwave irradiation affects the natural rubber (NR) phase of GTR more strongly than the styrene–butadiene rubber (SBR) phase. This behavior was attributed to the greater interaction of microwaves with conductive components such as carbon black.^29^ Formulation oils may also alter devulcanization behavior. Pistor et al. examined the influence of paraffinic oil on the network structure of waste EPDM during microwave-assisted recycling. Their findings showed that samples containing paraffinic oil experienced a lower temperature rise due to energy consumption during oil evaporation, and treatments exceeding 4 min result in material degradation.^13,26^

Importantly, increasing microwave intensity or exposure time beyond the optimum does not necessarily improve devulcanization; instead, it may induce random main-chain scission, oxidation, carbonization, radical recombination, and re-cross-linking rather than selective cleavage of sulfur cross-links. García et al. further showed through AFM and SEM analyses that excessive microwave exposure causes structural degradation that irreversibly deteriorates the thermal and mechanical integrity of the rubber while increasing the presence of carbonized residual particles.^22^ Consistent with this, Simon and Bárány,^30^ using Horikx-based analysis, demonstrated that although higher temperatures (180–200 °C) increase solubilization, they significantly reduce process selectivity and promote irreversible polymer degradation, whereas lower temperatures (140–160 °C) allow more selective devulcanization with less damage to the polymer backbone. Although many studies assess microwave treatment mainly through devulcanization efficiency, others have shown that it also affects surface chemistry, thermal stability, morphology, and physico-mechanical behavior, as reported by Kleps,^31^ Liu,^32^ Eom,^33^ and others. Bani et al. reported that effective devulcanization of EPDM initiates at temperatures above 300 °C and that the resulting by-products exhibited satisfactory physicochemical properties.^34^ Collectively, these studies show that the outcome of microwave treatment is highly sensitive to processing severity, and that increasing exposure does not necessarily lead to more effective network breakdown but may instead shift the material response toward network-forming reactions. A similar competition between chain scission and cross-link-forming reactions has been reported in studies on the thermo-oxidative aging of EPDM. In these systems, cross-link density decreases in the initial stage when chain scission is dominant and increases when radical recombination and secondary cross-linking become dominant.^35–37^ Although thermo-oxidative aging and microwave treatment involve different heating conditions and exposure times, the similarity in the observed network response provides a useful basis for interpreting the present results. Therefore, changes in cross-link density should be considered as the net result of competing scission and cross-link-forming reactions rather than as direct evidence of devulcanization alone.

Despite the growing interest in microwave-assisted rubber recycling, limited attention has been given to the combined influence of the particle-size fraction and exposure time on the response of highly filled industrial EPDM waste. Previous studies have generally focused on the overall devulcanization efficiency of bulk or unsieved ground rubber, whereas particle size-dependent heating and the competition between network-breaking and network-forming reactions have not been clearly distinguished in industrial EPDM compounds containing substantial amounts of carbon black, inorganic fillers, and process oil. It was hypothesized that the fine fraction and short microwave exposure would favor more uniform heating and net network loosening, whereas the coarse fraction and prolonged exposure would increase the likelihood of localized overheating, radical recombination, and secondary cross-link formation. To test this hypothesis, the effects of particle-size fraction and microwave exposure time were evaluated using apparent sol fraction and apparent cross-link density as comparative network indicators, supported by ATR-FTIR, TGA, XPS, and SEM analyses. The 60-treated fractions were subsequently incorporated into new formulations and revulcanized to assess their reprocessing performance through surface roughness, hardness, and tensile property measurements. This approach distinguishes the effects of particle size and treatment duration while linking the structural response of waste EPDM to the functional properties of the revulcanized compounds.

## Experimental Details

### Grinding, Particle Size Characterization, and Fractionation of Filled Industrial EPDM Waste

This study investigates the microwave treatment of filled industrial EPDM waste (w-EPDM) as a route for its potential recycling and reprocessing. The feedstock material consisted of industrial EPDM scraps generated during the rubber profile production process and supplied by a commercial manufacturing plant (Standard Profil - Düzce, Türkiye). The composition of the industrial EPDM waste is not disclosed in full due to proprietary restrictions; however, the compositional information can be reported as approximate values. The filled industrial EPDM compound, originally formulated for automotive sealing applications, contains approximately 20 ±2 wt % EPDM, 30 ±2 wt % carbon black, 20 ±2 wt % inorganic fillers, 20 ±2 wt % process oil, and 10 ±2 wt % small chemicals, including curing agents, activators, and processing aids. The rubber scraps were mechanically ground using a knife mill equipped with a nominal 100-mesh screen (150 μm).

These untreated, unsieved ground EPDM samples are referred to as g-EPDM. The particle-size distribution of g-EPDM was characterized using a particle size analyzer (HORIBA, Laser Scattering Particle Size Distribution LA-950) via the wet dispersion method using water as the dispersant to determine the full particle size distribution (Figure 1). The material showed a narrow distribution range with a volume-based mean diameter of 112.9 μm and a span value of 1.27. The characteristic cumulative sizes were determined as *D*_10_= 49.7 μm, *D*_50_ = 106.4 μm (median size), and *D*_90_ = 184.7 μm. This enabled the separation of fine (≤125 μm) and coarse (≥125 μm) fractions to evaluate the effect of particle size on microwave treatment response. The terms *“B”* and *“A”* indicate particle sizes below and above 125 μm, respectively (Table 1).

**Figure 1:**
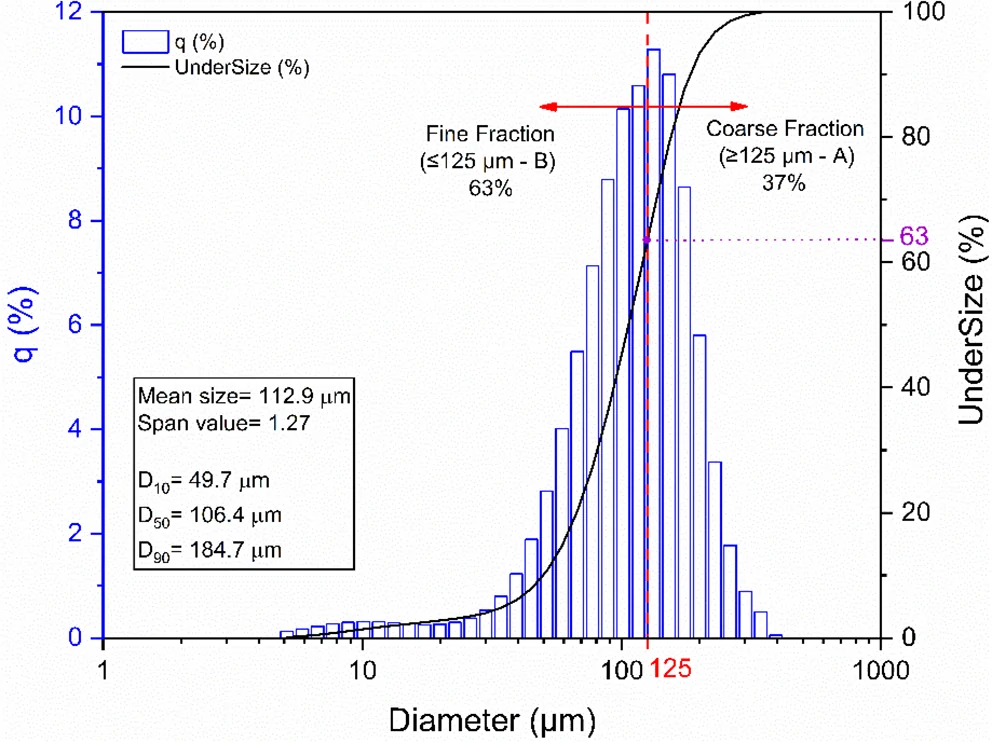
Particle size distribution of the unsieved ground EPDM powder (g-EPDM), showing both differential frequency distribution (*q*%) and the cumulative undersize curve. The vertical dashed line at 125 μm illustrates the physical separation threshold between the fine fraction (g-EPDM-B) and the coarse fraction (g-EPDM-A). The inset box displays the key statistical metrics, including mean size, span, *D*_10_, *D*_50_, and *D*_90_ values.

**Table 1:** Experimental Parameters for the Treatment at Constant Microwave Power (600 W) of Ground EPDM Waste, Including Particle Size, Exposure Duration, and Temperatures

sample name	particle size (μm)	exposure time (s)	temperature (°C)
g-EPDM	Unsieved (Broad distribution)^a^	N/A	N/A
d-EPDM-60-B	≤125 (Fine Fraction, B)	60	136
d-EPDM-120-B		120	145
d-EPDM-180-B		180	120
d-EPDM-60-A	≥125(Coarse Fraction, A)	60	125
d-EPDM-120-A		120	149
d-EPDM-180-A		180	156^b^

a 150 μm (100-mesh) represents the nominal size of the knife mill, which inherently produces a broad distribution containing both fine and coarse particles before sieving.

b A localized hot spot was observed.

### Microwave Treatment of Filled Industrial EPDM Waste

As a preparatory step prior to treatment and to investigate the effect of particle size, the ground EPDM was sieved using standard 120-mesh (125 μm) sieves according to ASTM D5644-01 (Retsch/AS 200 Basic, 20). Approximately 40 g of g-EPDM was placed in an open 600 mL glass beaker and distributed uniformly without compaction, resulting in an average powder-layer thickness of approximately 30 mm. The beaker was positioned at the center of the rotating glass turntable in a commercial microwave system operating at 2.45 GHz (Samsung). The magnetron power was set to 600 W, and exposure times of 60, 120, and 180 s were applied. The same sample mass, glass beaker, powder-layer thickness, and position within the microwave cavity were maintained for all treatments. No modification was made to the microwave cavity, and no internal mechanical stirring system was installed. Since ignition of the sample was observed at an exposure time of 240 s, the maximum treatment time was limited to 180 s. The treated EPDM samples are referred to as d-EPDM. The maximum surface temperature was measured using a thermal camera (Ti27, Fluke, USA). The camera-to-sample distance was kept constant during all measurements. Each treatment condition was performed once (*n* = 1). Treatment was performed at a constant microwave power, and the samples were differentiated based on exposure time (60 s, 120 s, 180 s) and particle size fraction.

### Evaluation of Network-Related Changes

The efficiency of EPDM waste microwave treatment was evaluated through sol fraction (%) and apparent cross-link density determination, conducted using the swelling test. Before performing the swelling test, the g-EPDMs and d-EPDMs were first immersed in acetone for 1 h to extract uncured low-molecular-weight additives. The powder samples were then oven-dried at 80 °C for 1 h, and the initial mass (*m*_0_) was recorded. Subsequently, approximately 1 g of each sample was placed in a cellulose cartridge and immersed in 350 mL of toluene at 100 °C for 24 h for extraction via the Soxhlet apparatus. After Soxhlet extraction, the samples were dried at 80 °C until constant mass, and the extracted dry mass was recorded (*m*_f_). The extracted powders were then immersed in fresh toluene in pre-weighed glass vials at room temperature for 72 h, with the solvent refreshed every 24 h. After excess surface toluene was removed, the vials were immediately capped, and the swollen mass was recorded as (*m*_s_). Finally, the swollen samples were dried again at 80 °C until constant mass, and the post-swelling dry mass was recorded (*m*_sf_). The sol fraction was then calculated using the equation provided below (eq 1)

1 $$sol\;fraction\;\left(\%\right)=\frac{\left(m_{0}-m_{f}\right)}{m_{0}}\times 100$$

The apparent cross-link density (ρ_c_) was calculated using the Flory–Rehner equation as follows:^38^

2 $$\rho_{c}=\frac{-\left[\ln\left(1-V_{r}\right)+V_{r}+XV_{r}^{2}\right]}{V_{s}\left(V_{r}^{1/3}-\frac{V_{r}}{2}\right)}$$

where ρ_*c*_, *V*_s_, *V*_r_, and χ represent apparent cross-link density (mol/cm^3^), molar volume of solvent (106.13 cm^3^/mol), the volume fraction of the polymer in the swollen specimen, and the polymer–solvent interaction parameter (0.49). The volume fraction of the polymer in the swollen g-EPDM and d-EPDM powder (*V*_r_) was calculated as follows

3 $$V_{r}=\frac{\frac{\mathrm{weig}ht\;\mathrm{of}\;\mathrm{dry}\;\mathrm{rubber}}{\mathrm{density}\;\mathrm{of}\;\mathrm{rubber}}}{\frac{\mathrm{weig}ht\;\mathrm{of}\;\mathrm{dry}\;\mathrm{rubber}}{\mathrm{density}\;\mathrm{of}\;\mathrm{rubber}}+\frac{\mathrm{weig}ht\;\mathrm{of}\;\mathrm{solvent}\;\mathrm{absorbed}\;\mathrm{by}\;\mathrm{sample}}{\mathrm{density}\;\mathrm{of}\;\mathrm{solvent}}}$$

Finally, the apparent devulcanization degree (DP) was calculated using the following equation

4 $$DP\left(\%\right)=\left\lceil 1-\left(\frac{c\mathrm{rosslink density of treated rubber}}{c\mathrm{rosslink density of untreated reference rubber}}\right)\right\rceil \times 100$$

It should be noted that the Kraus correction was not applied in this study because reliable sample-specific filler volume fractions could not be determined. Since grinding and particle-size fractionation may alter the filler distribution, the use of an assumed common filler content could introduce additional uncertainty. Therefore, the uncorrected values were used only for relative comparisons within the investigated material system and are reported as apparent cross-link density (apparent CLD) and apparent devulcanization percentage (apparent DP%). The apparent sol fraction and apparent cross-link density were determined once for each sample and were not subjected to inferential statistical analysis.

### Chemical and Surface Characterization

Attenuated Total Reflectance-Fourier Transform Infrared spectroscopy (ATR-FTIR, Thermo Scientific NICOLET iS10) was utilized to analyze the changes induced by the microwave treatment on functional groups and chemical bond types. The measurements were conducted using the Attenuated Total Reflectance (ATR) method, in which g-EPDM and d-EPDM samples were positioned on the crystal surface. Spectra were collected within the wavenumber range of 4000–650 cm^–1^ with 25 scans.

X-ray photoelectron spectroscopy (XPS, Thermo Scientific K-α) equipped with a monochromatic Al–Kα (1486.7 eV) was performed to determine the elemental composition and surface chemistry of g-EPDM and d-EPDM using a beam size of 400 μm diameter. Survey spectra were acquired using a pass energy of 150 eV, an energy step size of 1.0 eV, 15 scans, and a dwell time of 10 ms. High-resolution spectra were collected using a pass energy of 30 eV, an energy step size of 0.1 eV, 10 scans, and a dwell time of 50 ms. Before sample analysis, the instrument binding-energy scale was calibrated using the C 1s reference at 284.8 eV. No post-acquisition charge correction or rigid binding-energy shift was subsequently applied to the individual sample spectra, thereby retaining their as-acquired peak positions. The XPS spectra were processed and peak-fitted using Thermo Scientific Avantage software (version 5.9912). A Smart background was applied before peak fitting. Component peaks were represented using Gaussian–Lorentzian convolution functions with a fixed L/G mixing parameter of 30% and without an asymmetric tail contribution. Peak optimization was performed using the Powell algorithm, with the FWHM restricted to the range of 0.5–3.5 eV. The convergence tolerance and maximum number of iterations were set to 0.0001 and 110, respectively. Fitting quality was evaluated collectively from the agreement between the experimental spectrum and fitted envelope, the absence of systematic trends in the residual trace, minimization of the normalized chi-square value, and the degree to which the Abbe criterion approached unity.

### Thermal Analysis

Thermogravimetric Analyses (TGA) were conducted on a Perkin Elmer STA 6000 at a heating rate of 10 °C min^–1^. The experiments were performed from room temperature to 600 °C in an inert nitrogen atmosphere. To eliminate signal noise and clearly identify the maximum degradation temperatures (*T*_max_), the first derivative of the TG curves (dTG) was smoothed using the Savitzky–Golay method with a polynomial order of 2 and a window size of 150 points in *OriginPro software*.

### Morphological Analysis

Scanning electron microscopy (SEM, ZEISS GEMINI 560) was used for examination of the surface morphology of samples. The samples were placed onto carbon tape, and the excess material was removed by gentle air blowing and then coated with a thin layer of gold/palladium alloy before the observations in order to provide conductivity, which is essential for the SEM observations. The SEM images were acquired in secondary electron (SE) mode with 150× and 2000× magnifications at 10 kV to obtain the surface morphology.

### Preparation and Characterization of Revulcanized EPDM Compounds

The material used in this study was an ethylene-propylene-diene monomer (EPDM) compound, supplied by Standard Profil. Six formulations were prepared in separate single mixing batches by substituting 10 phr EPDM in the EPDM compound with untreated ground EPDM (C-g-EPDM, C-g-EPDM-B, and C-g-EPDM-A) or microwave-treated EPDM obtained under the selected treatment conditions (C-d-EPDM-60-B and C-d-EPDM-60-A). The virgin EPDM sealing compound was used as the reference and denoted as C-EPDM. All compounds were mixed in a lab-scale mixer (Intermix Mixer, Carter Bros 1.5 L) for 5 min under the same mixing conditions (47 rpm motor speed) while keeping the amounts of the other compounding ingredients constant. After mixing, the compounds were passed through the lab-scale cylinder. Subsequently, the prepared compounds were molded into plates in a compression press at 180°C for 7.5 min.

The surface roughness and hardness measurements were performed on the sheet samples to evaluate the effect of the recycled EPDM phase on the surface characteristics and hardness of the revulcanized materials. Surface roughness measurements were performed using a Mitutoyo SurfTest SJ-301 profilometer. The measurements were conducted according to the profile method described in ISO 4287 and ISO 4288,^39,40^ in line with the updated terminology of ISO 21920-2:2021.^41^ The measuring distance was 4.0 mm, and the cut length was 0.8 mm. For each formulation, five spatial measurements were obtained at different locations on the same plate (*n* = 5) and in the same measurement direction. The results were reported as mean ± standard deviation. The arithmetic mean roughness (*R*_a_) and mean roughness depth (*R*_*z*_) were calculated from the measured profiles. In addition, Shore A hardness was likewise measured at five different locations on each plate (*n* = 5) in accordance with DIN ISO 7619-1.

Tensile strength and elongation at break were determined using type 2 dumbbell specimens according to ISO 37 on a SHIMADZU AG-IS universal test machine. The tests were conducted at a crosshead speed of 500 mm/min under ambient laboratory conditions (23 ± 2 °C) using a 250 kN load cell. Three valid specimens were evaluated for each formulation (*n* = 3 specimen replicates), and the results were reported as mean ± standard deviation.

Statistical analyses were conducted using OriginPro 2018 (OriginLab Corporation, Northampton, MA, USA). Tensile strength and elongation at break were compared among the six formulations using one-way ANOVA. When the one-way ANOVA indicated a significant difference, pairwise comparisons were performed using Tukey’s honestly significant difference (HSD) test. Two-way ANOVA was used to determine the main and interaction effects of particle size and microwave treatment (untreated or 60 s) on tensile strength, elongation at break, *R*_a_, and *R*_*z*_ for the matched fine and coarse fractions. Statistical significance was defined as *p < 0.05*. Because each formulation was prepared as a single mixing batch, the tensile results represent specimen-level variability within that batch, whereas the roughness and hardness results represent spatial variability within one plate. These measurements do not represent independent production-batch replicates.

The factor-level coding, individual tensile-strength and elongation-at-break measurements, engineering stress–strain curves for all tested specimens and representative formulations, detailed one- and two-way ANOVA results, Tukey HSD pairwise comparisons, individual surface-roughness measurements, and corresponding two-way ANOVA results are provided in Tables S1–S7 and Figures S1–S2 of the Supporting Information.

## Results and Discussion

### Network Changes in Microwave-Treated EPDM Waste

The surface-temperature results reported in Table 1 did not follow a strictly monotonic trend with exposure time. The lower surface temperature measured for d-EPDM-180-B (120 °C) than for d-EPDM-120-B (145 °C) may be associated with non-uniform heating of the unstirred powder samples. Previous studies have shown that stirring can improve temperature uniformity during the microwave treatment of ground rubber, whereas insufficient mixing may result in localized hot and cold regions.^30,42^ Since the thermal camera measured only the sample surface, the recorded value may not represent the maximum temperature reached within the powder.

The apparent sol fraction, apparent cross-link density, and apparent devulcanization degree of untreated and treated EPDM samples are summarized in Table 2. The apparent DP % values of the microwave-treated samples were calculated relative to the common unsieved g-EPDM reference. To evaluate microwave-induced changes independently of the initial fractionation response, the apparent sol fraction and apparent cross-link density of each treated sample were also compared directly with those of its corresponding untreated particle-size fraction. The observed increase in the soluble fraction, together with a decrease in apparent cross-link density, indicates net loosening of the three-dimensional rubber network. However, this response cannot be attributed solely to selective devulcanization, because selective cleavage, main-chain scission, and oxidative reactions may occur simultaneously. Conversely, a decrease in sol fraction together with an increase in apparent cross-link density indicates the formation of a less extractable and more restricted structure.^43^ Since the Kraus correction was not applied, the apparent cross-link density values may also include filler-related restrictions on swelling, particularly when the fine and coarse fractions are compared. Therefore, the apparent cross-link density and apparent devulcanization degree were used to follow relative trends and should be regarded as comparative network-change indicators rather than absolute measures of cross-link density or selective devulcanization.

**Table 2:** Apparent Sol Fraction, Apparent Cross-Link Density, and Apparent Devulcanization Degree of Untreated Unsieved, Untreated Size-Fractionated, and Microwave-Treated EPDM Samples

sample name	apparent sol fraction (%)	apparent cross-link density x 10^–4^ (mol/cm^3^)	apparent devulcanization degree (%)
g-EPDM	23.45	0.87	N/A
g-EPDM-B	20.07	0.84	N/A
g-EPDM-A	21.82	0.92	N/A
d-EPDM-60-B	25.39	0.72	17.11
d-EPDM-120-B	20.67	0.82	5.86
d-EPDM-180-B	18.32	0.94	–7.64
d-EPDM-60-A	23.47	0.84	3.49
d-EPDM-120-A	18.68	1.05	–19.83
d-EPDM-180-A	17.52	1.24	–42.21

The untreated, unsieved g-EPDM showed an apparent sol fraction of 23.45% and an apparent cross-link density of 0.87 × 10^–4^ mol/cm^3^. Size fractionation itself changed the swelling response: g-EPDM-B showed an apparent sol fraction of 20.07% and an apparent cross-link density of 0.84 × 10^–4^ mol/cm^3^, whereas g-EPDM-A showed corresponding values of 21.82% and 0.92 × 10^–4^ mol/cm^3^. Within the fine fraction, 60 s microwave treatment increased the apparent sol fraction from 20.07% to 25.39% and decreased the apparent cross-link density from 0.84 × 10^–4^ to 0.72 × 10^–4^ mol/cm^3^. Within the coarse fraction, the corresponding treatment increased the apparent sol fraction from 21.82% to 23.47% and decreased the apparent cross-link density from 0.92 × 10^–4^ to 0.84 × 10^–4^ mol/cm^3^. Thus, the 60 s treatment produced a net network-loosening response relative to the matching untreated fraction in both particle-size groups, with the larger change observed in the fine fraction. Among all samples, d-EPDM-60-B exhibited the highest apparent sol fraction (25.39%) and the lowest apparent cross-link density (0.72 × 10^–4^ mol/cm^3^), corresponding to an apparent devulcanization degree of 17.11% relative to the untreated ground material. Nevertheless, the swelling measurements do not identify which chemical bonds were affected. Selective sulfur-cross-link cleavage may have contributed to the observed response, but possible contributions from nonselective chain scission, oxidation, and changes in the extractable phase cannot be excluded. The different responses of the two fractions may be related to particle-size-dependent differences in microwave heating and heat dissipation^14^

As the exposure time was prolonged, the apparent devulcanization degree calculated relative to unsieved g-EPDM decreased. Within the fine fraction, the apparent cross-link density increased from 0.72 × 10^–4^ mol/cm^3^ at 60 s to 0.82 × 10^–4^ mol/cm^3^ at 120 s and 0.94 × 10^–4^ mol/cm^3^ at 180 s, compared with 0.84 × 10^–4^ mol/cm^3^ for untreated g-EPDM-B. Thus, the network-loosening response was strongest at 60 s, became limited at 120 s, and shifted toward a more restricted apparent network at 180 s. The same progression was more pronounced in the coarse fraction: compared with 0.92 × 10^–4^ mol/cm^3^ for untreated g-EPDM-A, the apparent cross-link density was 0.84 × 10^–4^ mol/cm^3^ at 60 s, 1.05 × 10^–4^ mol/cm^3^ at 120 s, and 1.24 × 10^–4^ mol/cm^3^ at 180 s. Accordingly, only the 60 s treatment decreased the apparent cross-link density relative to the matching untreated coarse fraction, whereas the longer treatments increased it.

Negative apparent devulcanization degree values indicate that the apparent cross-link density after microwave treatment is higher than that of the common unsieved g-EPDM reference. These values should not be interpreted as a mechanistic measure of “negative devulcanization”, but rather as evidence that network-restricting effects became more pronounced under these conditions. Such a response may arise from radical recombination, secondary cross-link formation, oxidation, and other treatment-induced structural changes. The same competition between chain scission and cross-link formation has been reported during EPDM aging.^35,37^ When the localized heat generated by carbon black exceeds the thermal dissipation limits of the matrix (thermal runaway), the resulting reactive species may undergo recombination instead of producing soluble chain fragments.^24^ The decrease in apparent sol fraction together with the increase in apparent cross-link density of 120 and 180 s is therefore consistent with a shift in the net response toward network-forming reactions. This shift was more pronounced in the coarse fraction.

### Chemical Structure Changes of Microwave-Treated EPDM Waste

Figure 2 shows ATR-FTIR spectra of the microwave-treated EPDM samples recorded in the range of 4000–650 cm^–1^. The main transmittance bands observed at approximately 2925 cm^–1^ and 2853 cm^–1^ correspond to the asymmetric and symmetric C–H stretching vibrations of the CH_2_ and CH_3_ groups, respectively, while the bands around 1460 and 1370 cm^–1^ correspond to C–H bending vibrations.^44^ Importantly, the characteristic transmittance bands of the aliphatic EPDM backbone exhibited no significant shift in peak positions compared to the untreated sample, g-EPDM. This suggests that the major aliphatic backbone signatures remain broadly unchanged after microwave treatment.

**Figure 2:**
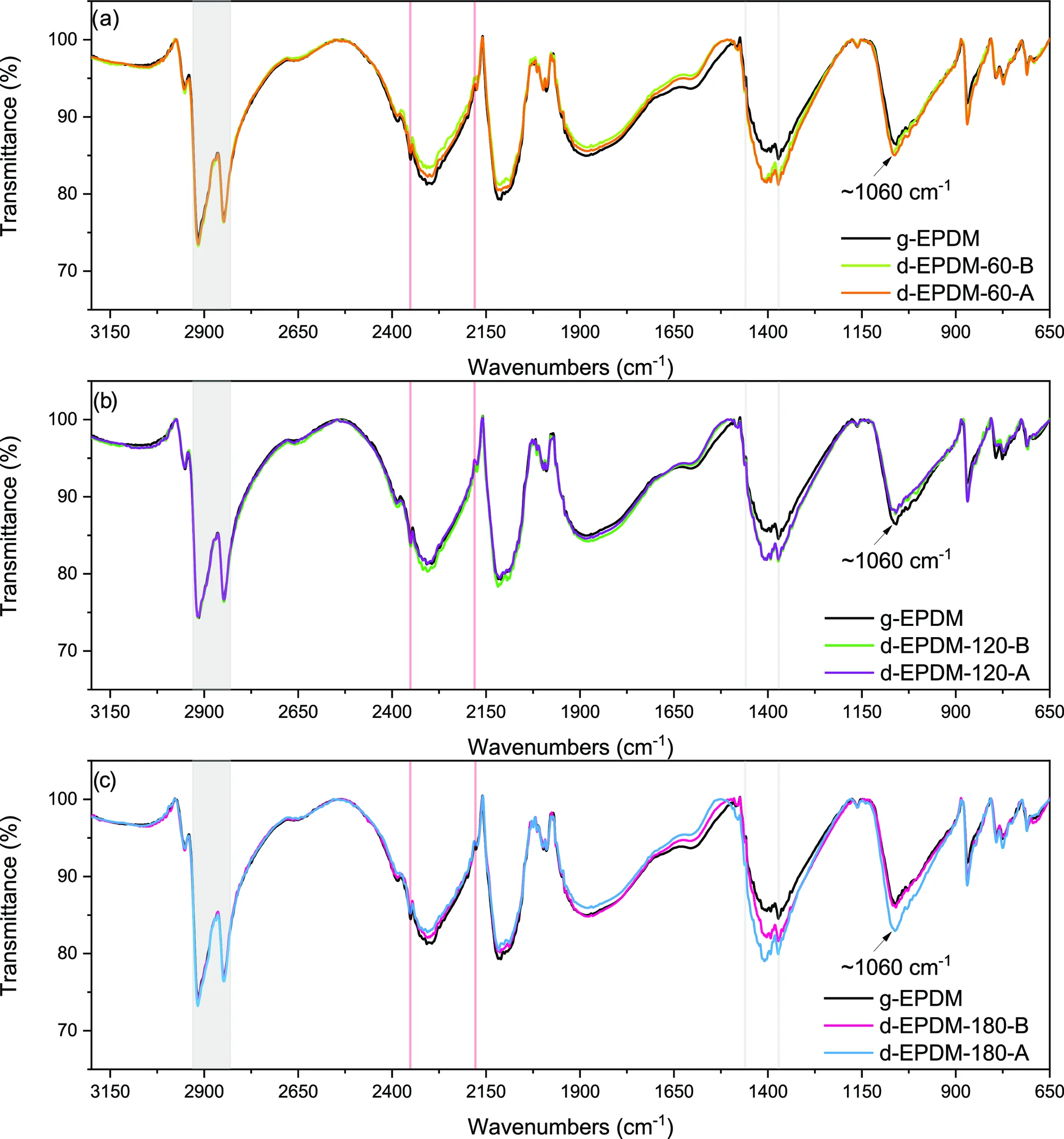
ATR-FTIR spectra of untreated, g-EPDM, and treated samples, d-EPDM, under 600 W microwave power at different exposure times: (a) 60 seconds, (b) 120 seconds, and (c) 180 seconds.

However, variations in transmittance intensity were observed with different treatment parameters, suggesting changes in the local chemical environment and effective concentration of infrared-active groups. During microwave treatment, the supplied thermal energy may increase the susceptibility of sulfur-containing bonds to cleavage because the bond energies of S–S (225 kJ/mol) and C–S (260 kJ/mol) are lower than that of the C–C backbone (350 kJ/mol).^45,46^ However, this comparison provides only a thermodynamic rationale and does not, by itself, demonstrate selective S–S or C–S bond cleavage under microwave treatment. Experimental studies have shown that the resulting network response is also governed by the final treatment temperature, exposure conditions, and filler-mediated energy absorption, and that cross-link cleavage may occur concurrently with new bond formation and polymer-chain degradation.^24,27,47^ Horikx-based analyses have further shown that microwave-treatment selectivity can decrease at higher temperatures even when the measured cross-link density is reduced.^47^ Therefore, particularly because the spectral range did not include the typical S–S vibration region (∼485 cm^–1^), the ATR-FTIR results are interpreted as evidence of network modification rather than direct proof of sulfur-bond scission or selective devulcanization.

A broad band was observed at approximately 1060 cm^–1^ in all spectra. Considering the high inorganic-filler content of the industrial EPDM compound, this band was primarily assigned to Si–O stretching vibrations associated with silica- or silicate-containing fillers.^44,48,49^ Treatment-dependent changes in its intensity and broadening may also include an overlapping contribution from S–O vibrations.^12,24^ However, because Si–O and S–O absorptions cannot be distinguished in this spectral region by ATR-FTIR alone, the band was not interpreted as direct evidence of sulfoxide or sulfone formation or sulfur-cross-link oxidation.

On the basis of these results, the 60 s treatment was selected for subsequent TGA, XPS, SEM, and revulcanization studies because it was the only exposure time that decreased the apparent cross-link density relative to the corresponding untreated fraction in both particle-size groups. Longer exposure times did not meet this selection criterion and were therefore not included in subsequent detailed characterization. Accordingly, the findings from these analyses are limited to the 60 s of treatment and are not extrapolated to longer exposure times.

### Surface Chemical Changes after Microwave Treatment

X-ray photoelectron spectroscopy (XPS) was employed to investigate the surface chemical changes induced by microwave treatment of EPDM (Figure 3). XPS is a surface-sensitive technique and, therefore, was used here to evaluate how the outermost surface chemistry of the EPDM particles changed after microwave exposure. The survey spectra revealed the presence of O 1s, C 1s, and S 2p core-level peaks along with Zn 2p and Ca 2p signals originating from the filler components in the filled EPDM compound.

**Figure 3:**
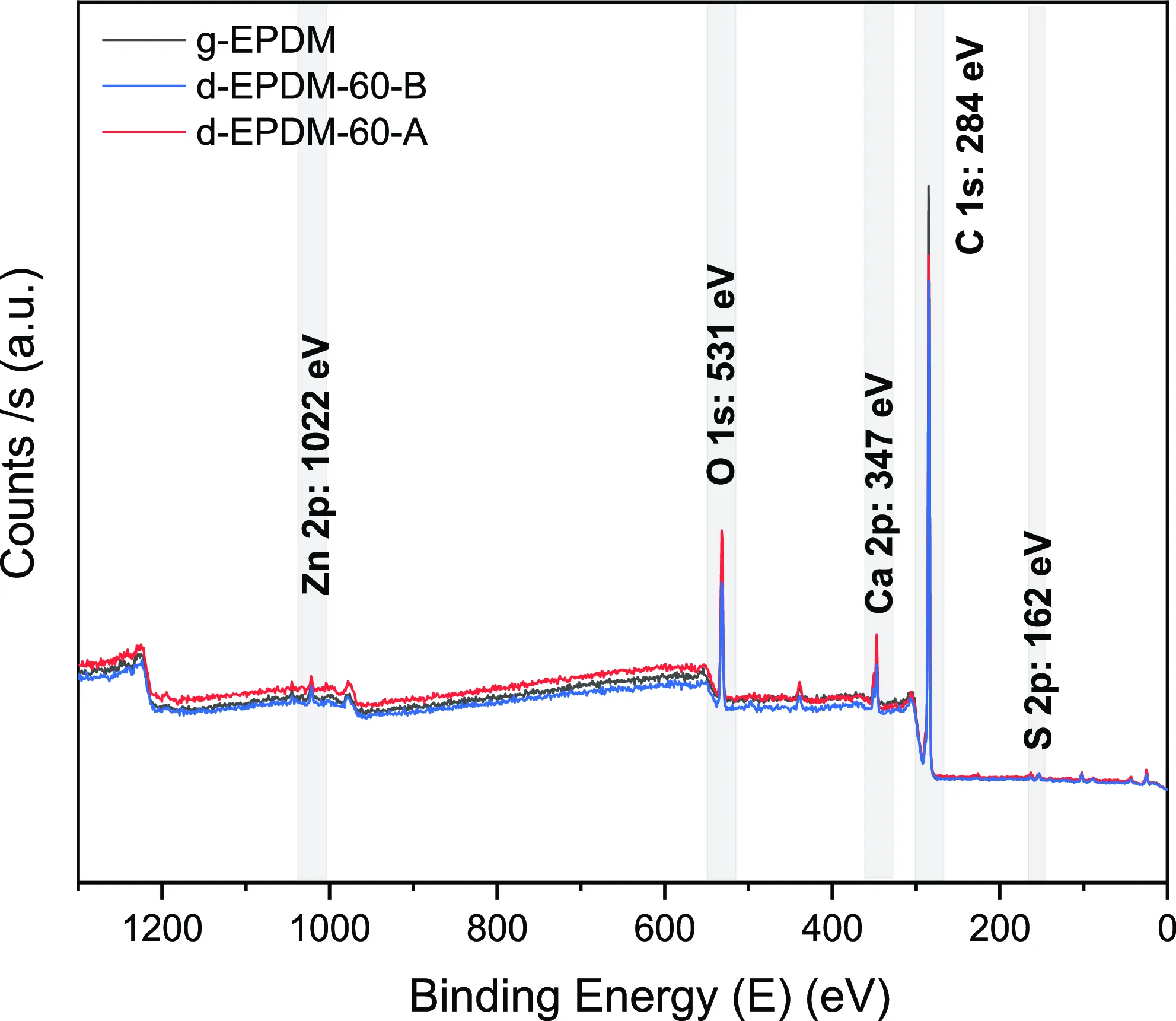
XPS survey spectra of untreated (g-EPDM) and microwave-treated (d-EPDM-60-B and d-EPDM-60-A) samples.

To further elucidate the surface chemical composition, high-resolution XPS core-level spectra of the C 1s and S 2p regions were acquired. The identified elements, peak positions (binding energies, BE), and corresponding relative fitted contributions for the untreated and microwave-treated EPDM samples are summarized in Table 3. The high-resolution C 1s spectra (Figure 4a–c) were fitted using three-component peaks. The dominant component peak at ∼284 eV is assigned to C–C/C=C, while the component at ∼285 eV is commonly associated with C–S bonding. The higher-binding-energy component peak at ∼288 eV is attributed to oxidized carbon types, specifically C=O groups.^50,51^ After microwave treatment, the relative contribution of C–C/C=C increased from 70.19% in g-EPDM to 80.88% in d-EPDM-60-B and 77.88% in d-EPDM-60-A, indicating that the particle surface became relatively richer in hydrocarbon-type carbon environments after microwave exposure. Correspondingly, the C–S component decreased from 24.16% to approximately 13%, suggesting a reduction in sulfur-containing carbon environments at the surface. The increase in the C=O fraction, particularly in d-EPDM-60-A (5.16%), may point to enhanced surface oxidation under more intense localized heat accumulation. Notably, the high-resolution S 2p spectra of g-EPDM (Figure 4d–f) exhibited only a broad contribution assigned to S–S/C–S environments (∼163.25 eV, 1.69%), whereas the microwave-treated samples showed an additional broad contribution assigned to oxidized sulfur species containing S–O bonds (∼168 eV).^52,53^ The relative S–O contributions were 0.76% for d-EPDM-60-B and 0.94% for d-EPDM-60-A, showing a higher relative contribution of oxidized sulfur-containing species at the particle surface after microwave treatment. While the S–S/C–S content slightly decreased in d-EPDM-60-B (1.63%), it increased to 2.22% in d-EPDM-60-A, indicating a difference in the relative abundance of sulfur surface environments detected at the particle surface.

**Figure 4:**
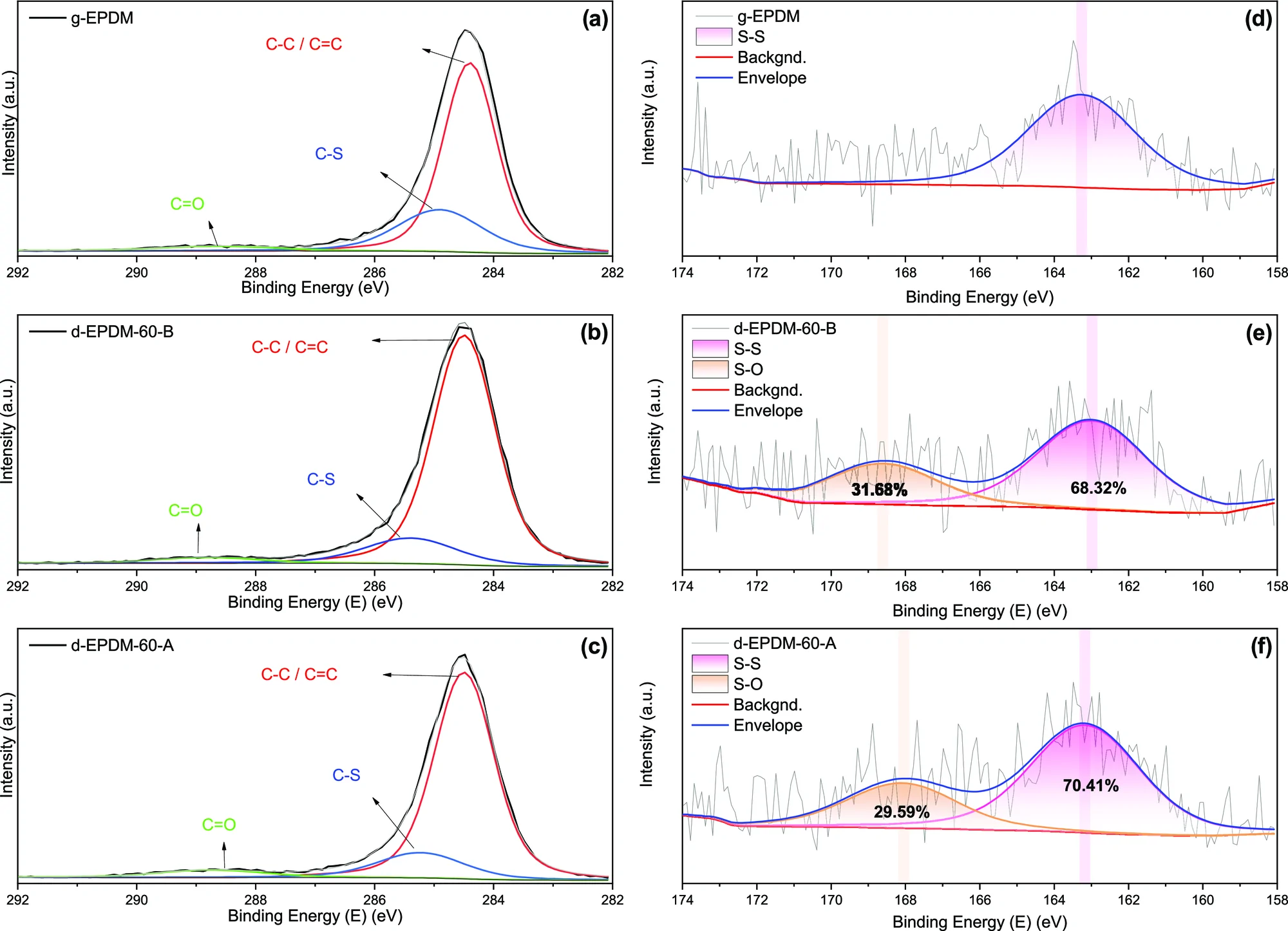
High-resolution XPS spectra and corresponding peak-fitting results for untreated and microwave-treated EPDM samples: (a–c) C 1s regions and (d–f) S 2p regions. Percentages shown in the S 2p panels represent the relative fitted-area contributions within the corresponding S 2p regions.

**Table 3:** XPS Peak Positions (Binding Energy, BE) and Relative Fitted Contributions for Untreated and Microwave-Treated EPDM Samples

		g-EPDM		d-EPDM-60-B		d-EPDM-60-A	
core level	structure	peak BE	relative contribution (%)	peak BE	relative contribution (%)	peak BE	relative contribution (%)
S 2p	S–S/C–S	163.25	1.69	162.98	1.63	163.16	2.22
	S–O			168.59	0.76	168.09	0.94
C 1s	C–C/C=C	284.39	70.19	284.49	80.88	284.50	77.88
	C–S	284.91	24.16	285.40	13.32	285.23	13.8
	C=O	288.65	3.96	288.81	3.41	288.74	5.16

Overall, these findings indicate that microwave treatment changed the relative contributions of sulfur-containing surface environments detected at the particle surface. The increased relative contribution of the C=O component is also consistent with some degree of surface oxidation.^54–57^ ATR-FTIR spectra showed no noticeable shifts in the main aliphatic C–H bands, whereas XPS revealed a lower contribution from C–S-related carbon environments and the presence of oxidized sulfur species after microwave treatment. Considering the surface-sensitive nature of XPS, these observations are interpreted as surface chemical changes, whereas the bulk network response was evaluated separately using the apparent sol fraction and apparent cross-link density results.

### Thermal Behavior of Microwave-Treated EPDM Waste

The thermal properties of the untreated, unsieved EPDM (g-EPDM), untreated size-classified fraction (g-EPDM-B and g-EPDM-A), and their corresponding microwave-treated samples (d-EPDM-60-B and d-EPDM-60-A) were investigated to distinguish the effects of particle-size fraction and microwave treatment on thermal degradation. The thermogravimetric (TG) and derivative thermogravimetric (DTG) curves of g-EPDM and treated samples are presented in Figure 5. The g-EPDM is mainly composed of four primary constituents: the EPDM polymer phase, carbon black, inorganic fillers, and process oil. According to Zanchett et al.^29^ the thermal degradation of EPDM typically proceeds through successive stages: beginning with the evaporation of volatile compounds and softeners at low temperature, followed by the decomposition of the polymer phase at higher temperatures. Under oxidative conditions, subsequent mass-loss steps would include the combustion of carbon black, whereas inorganic fillers such as metal oxides would persist as nonvolatile residue. In the present study, since no oxidative atmosphere was applied and the analysis was terminated at 600 °C, carbon black oxidation did not occur, and the inorganic fillers also remained in the residue. Consequently, the residual mass represents the combined nonvolatile fraction and does not allow the individual filler contributions or filler segregation between the particle-size fractions to be determined.

**Figure 5:**
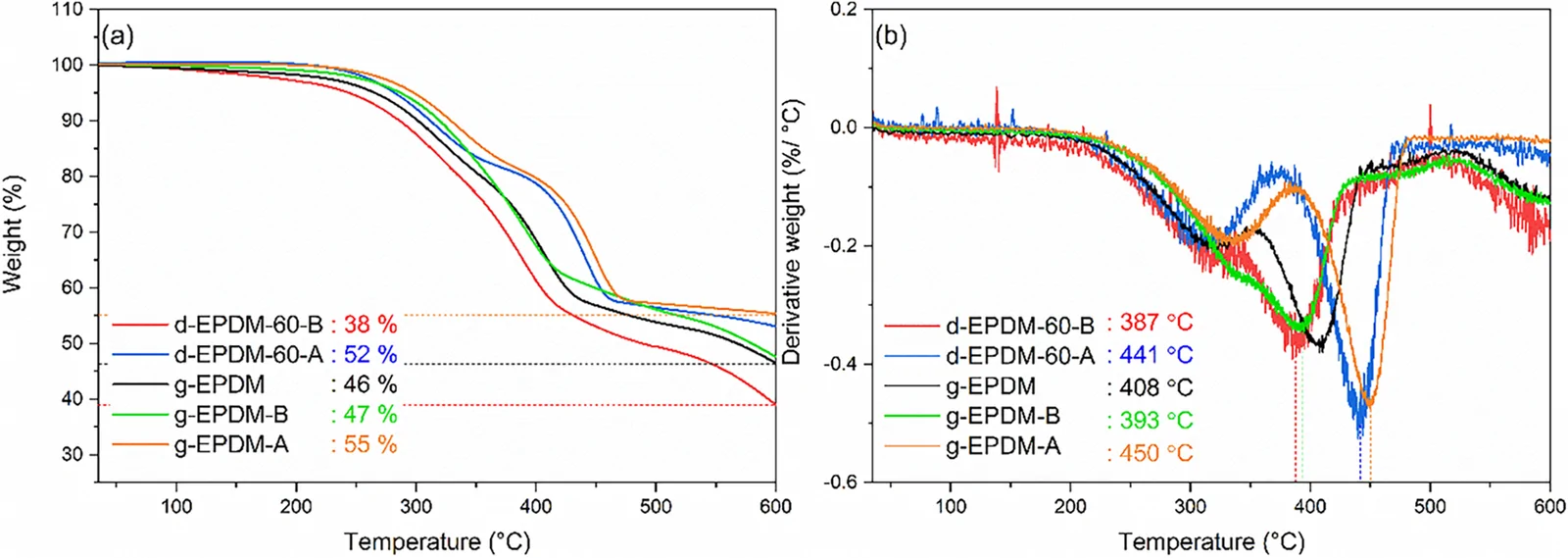
Comparative thermogram (a) and derivative weight results (b) of untreated g-EPDM, g-EPDM-B, g-EPDM-A, d-EPDM-60-B, and d-EPDM-60-A. The vertical dashed lines indicate the main maximum degradation temperatures (*T*_max_), and the horizontal dashed lines indicate the residual mass values at 600 °C.

As shown in Figure 5a and 5b, all samples exhibited a characteristic two-step mass-loss region under nitrogen. The first step at ∼320 °C is mainly associated with the decomposition of volatile compounds, oil, and softener, while the second and major step likely represents the thermal decomposition of the EPDM polymer backbone and its network structure.^58^ The untreated size-classified samples showed distinctly different thermal degradation profiles. The main maximum degradation temperature (*T*_max_) of g-EPDM-B occurred at approximately 393 °C, whereas that of g-EPDM-A occurred at approximately 450 °C; the unsieved g-EPDM exhibited an intermediate value of approximately 408 °C. The residual mass at 600 °C was higher for g-EPDM-A (approximately 55%) than for both g-EPDM-B (approximately 47%) and unsieved g-EPDM (approximately 46%).

The difference between the untreated fractions may be related to particle-size-dependent heat transfer processes. An increase in particle size may promote the development of a temperature gradient between the particle surface and core, thereby increasing resistance to internal heat transfer and to the release of volatile degradation products.^14^ In addition, Lewandowski et al.^46^ investigated cryogenically ground and size-classified ground tire rubber and reported that the fine fraction (<63 μm) contained a high carbon black content of 52 wt %. Their results suggest that grinding and sieving may separate the waste rubber not only according to particle size but also according to the relative proportions of rubber and filler components. Liang et al. also demonstrated the use of TGA under oxidative conditions to characterize the carbon black and inorganic fractions of recycled EPDM powder.^59^ The different residual masses of g-EPDM-B and g-EPDM-A may reflect fraction-dependent compositional differences; however, because no independent compositional analysis was performed for the two particle-size fractions, this interpretation remains a possible explanation rather than evidence of filler segregation.

An analysis of the DTG curve for the d-EPDM-60-B sample showed that the maximum degradation temperature (*T*_max_) shifted to approximately 387 °C, compared with approximately 393 °C for the corresponding untreated g-EPDM-B. The residual mass also decreased from approximately 47% for g-EPDM-B to 39.03% for d-EPDM-60-B. Likewise, when compared with the corresponding untreated coarse fraction, g-EPDM-A, the *T*_max_ of d-EPDM-60-A was slightly lower, decreasing from approximately 450 to 441 °C. The residual mass also decreased slightly from approximately 55% for g-EPDM-A to 52% for d-EPDM-60-A. Within both particle-size fractions, microwave treatment produced a moderate shift of the main degradation peak toward lower temperatures. This shift indicates that microwave exposure altered the subsequent thermal decomposition behavior of the EPDM waste. In the fine fraction, the lower *T*_max_ occurred together with the lower apparent cross-link density reported in Table 2. However, *T*_max_ is not governed by cross-link density alone and may also be influenced by the overall compound composition, including the carbon black and inorganic fractions, possible thermal-oxidative and network-structural changes resulting from competing degradation and cross-linking reactions, and particle-size-dependent heat transfer process.^14,59–61^ Therefore, the decrease in *T*_max_ is considered consistent with, but does not independently demonstrate, a net network-loosening response. The smaller shifts observed in the coarse fraction indicate a more limited change in its measured thermal response.

Overall, the TG and DTG results show that both the initial particle size fraction and the microwave treatment influenced the thermal response of the EPDM waste. The pronounced difference between the untreated A and B fractions indicates a fraction-dependent thermal response under the tested conditions, while comparisons within the same fraction suggest that microwave treatment was accompanied by moderate shifts toward lower degradation temperatures.

### Surface Morphology of Untreated and Treated EPDM Waste

The surface morphologies of the untreated and microwave-treated EPDM powder samples examined by SEM are presented in Figure 6. Low-magnification SEM images of the untreated, unsieved EPDM powder, g-EPDM, showed irregularly shaped agglomerated particles with a broad size distribution (Figure 6a). After size classification, the fine fraction, g-EPDM-B, showed smaller and more separated particles, whereas the coarse fraction, g-EPDM-A, consisted of larger agglomerated particles retained on the 125 μm sieves Figure 6e, (i). At higher magnification, the untreated samples exhibited the heterogeneous structure typical of highly filled rubber compounds, including irregular surfaces and embedded inorganic filler-rich regions highlighted by white dashed circles (Figure 6b,f,j). After swelling and drying, the g-EPDM sample (Figure 6c) showed a relatively more open and loosened morphology. This change may be attributed to the removal of extractable low-molecular-weight components such as process oil, while swelling in toluene. The resulting increase in surface irregularity and partial removal of the soluble phase made sharp-edged filler particles and filler–rubber interfaces more visible in some regions, as indicated by the white dashed circles.

**Figure 6:**
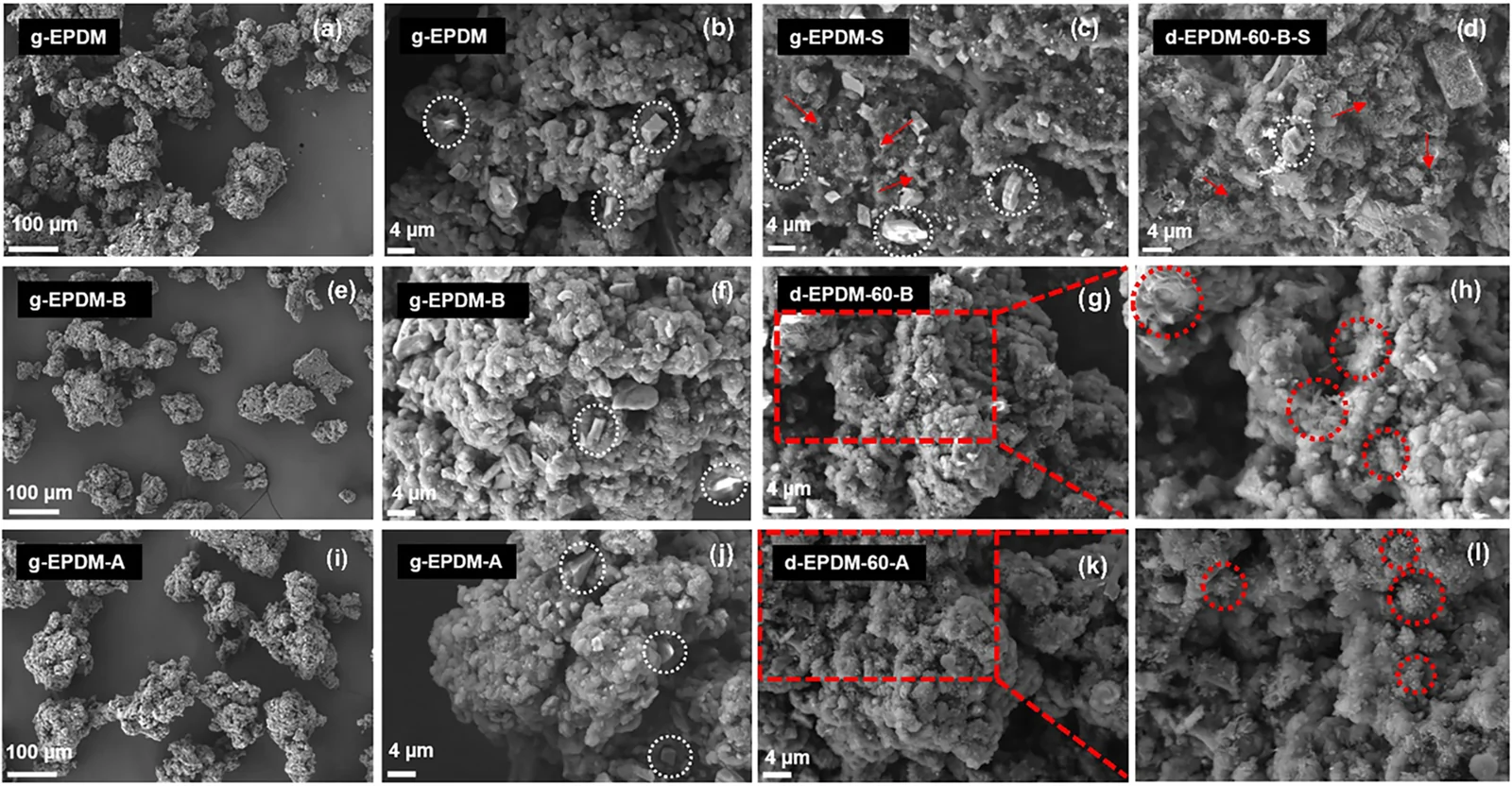
SEM micrographs of untreated and microwave-treated powder samples. (a, b) Untreated, unsieved EPDM powder, g-EPDM, at low and high magnification; (c) post-swelling and dried untreated EPDM powder, g-EPDM-S; (d) post-swelling and dried microwave-treated fine fraction, d-EPDM-60–B-S; (e, f) untreated fine fraction, g-EPDM-B, at low and high magnification; (g, h) microwave-treated fine fraction, d-EPDM-60-B, and the corresponding higher-magnification view of the selected region; (i, j) untreated coarse fraction, g-EPDM-A, at low and high magnification; and (k, l) microwave-treated coarse fraction, d-EPDM-60-A, and the corresponding higher-magnification view of the selected region. The suffix “S” refers to the samples examined after swelling and subsequent drying.

After microwave treatment, both the treated fine fraction (d-EPDM-60-B) and the treated coarse fraction (d-EPDM-60-A) exhibited localized increases in surface irregularity compared with their untreated counterparts (Figure 6g,k). Higher-magnification SEM images of the selected regions are presented in Figure 6h,l. Needle-like and flake-like structures were observed on the surface of the treated fine fraction, d-EPDM-60-B (Figure 6h). In contrast, predominantly nodular structures were observed on the surface of the treated coarse fraction, d-EPDM-60-A (Figure 6l). These representative morphological features are highlighted with red dashed circles. The observations qualitatively suggest that microwave exposure modified the surface morphology of the EPDM waste particles.^43,62–65^

When microwave treatment was followed by swelling in toluene (Figure 6d), additional changes in the surface morphology became evident. The increased visibility of the dark regions (indicated with red arrows) suggested the development of a loose and more porous structure, accompanied by a rougher surface after toluene exposure. Rather than directly proving devulcanization, these morphological features are consistent with the alteration of network and structural morphology after treatment. The surface features produced by the microwave treatment may have slightly increased the accessible surface area. This could contribute to the lower *T*_max_ values of the microwave-treated samples compared to their untreated counterparts.

### Surface Roughness and Hardness of Revulcanized EPDM Compounds

The surface roughness and hardness results of revulcanized sheet samples are summarized in Table 4. The reference compound, C-EPDM, showed the smoothest surface, with the lowest *R*_a_ (0.42 μm) and *R*_*z*_ (2.69 μm) values. This indicates a more uniform and homogeneous surface topography. The surface roughness of the compound containing untreated, unsieved EPDM powder, C-g-EPDM, increased to *R*_a_ = 0.66 μm and *R*_*z*_ = 4.49 μm. This result shows that the incorporation of the untreated, unsieved powder produced a more irregular surface than the reference. Among the untreated size-fractionated samples, the fine fraction, C-g-EPDM-B, showed lower *R*_a_ and *R*_*z*_ values (0.57 and 3.77 μm) than the coarse C-g-EPDM-A fraction (0.61 and 4.16 μm). Microwave treatment caused only small changes within both particle-size groups. For the fine fraction, *R*_a_ and *R*_*z*_ changed to 0.60 and 3.82 μm, while the corresponding values for the treated coarse fraction were 0.63 and 4.18 μm.

**Table 4:** Surface Roughness Parameters (*R*_a_ and *R*_*z*_) and Hardness Values of the Revulcanized EPDM Compounds Containing Untreated and Microwave-Treated Recycled EPDM

	*R*_a_ (μm)	*R*_*z*_ (μm)	hardness (Shore A)
C-EPDM	0.42 ± 0.03	2.69 ± 0.19	75.8 ± 0.84
C-g-EPDM	0.66 ± 0.06	4.49 ± 0.39	75.2 ± 0.84
C-g-EPDM-B	0.57 ± 0.10	3.77 ± 0.51	75.4 ± 1.14
C-d-EPDM-60-B	0.60 ± 0.04	3.82 ± 0.30	75.2 ± 1.10
C-g-EPDM-A	0.61 ± 0.05	4.16 ± 0.41	74.6 ± 0.89
C-d-EPDM-60-A	0.63 ± 0.09	4.18 ± 0.35	75.6 ± 0.89

Two-way ANOVA showed no significant effects of particle size, microwave treatment, or their interaction on R_a_ (all *p* values >0.05). For *R*_*z*_, the particle size effect was close to the significance level but was not statistically significant (*F*(1,16) = 4.427, *p* = 0.052). Microwave treatment and the interaction had no significant effects on Rz (p > 0.05). The results, therefore, show a particle-size-related tendency for R*_z_*, whereas *R*_a_ remained comparable among the size-fractionated formulations. The unsieved formulation was not included in the factorial analysis. Its higher mean roughness, together with the agglomerated domains observed by SEM, may be related to its broad particle size distribution and irregular particle morphology.^26,66^

In contrast, hardness remained almost constant for all revulcanized compounds (74–75 Shore A). These results show that at a loading of 10 phr, the presence and treatment form of the recycled EPDM had only a minor effect on the macroscopic rigidity of the revulcanized compounds. Overall, surface roughness showed greater numerical variation than hardness; however, no statistically significant main or interaction effects were identified for *R*_a_, and the particle size effect on *R*_*z*_ only approached significance.

### Tensile Properties of Revulcanized EPDM Compounds

The tensile strength and elongation at break results of the revulcanized compounds are presented in Figure 7 and Table 5. The reference compound, C-EPDM, showed a tensile strength of 8.21 ± 0.26 MPa, while C-g-EPDM, containing untreated and unsieved EPDM powder, showed 7.78 ± 0.25 MPa. The untreated fine and coarse fractions exhibited similar tensile strength values. Microwave treatment caused only a slight change in the coarse fraction, from 7.75 ± 0.07 to 7.86 ± 0.11 MPa, whereas the fine fraction increased from 7.71 ± 0.17 to 8.32 ± 0.34 MPa. C-d-EPDM-60-B therefore showed the highest mean tensile strength. All formulations remained above the reported target value for automotive sealing systems (> 7.0 MPa),^67^ showing that the replacement of 10 phr virgin EPDM with recycled EPDM maintained the tensile strength above this application-related target.

**Figure 7:**
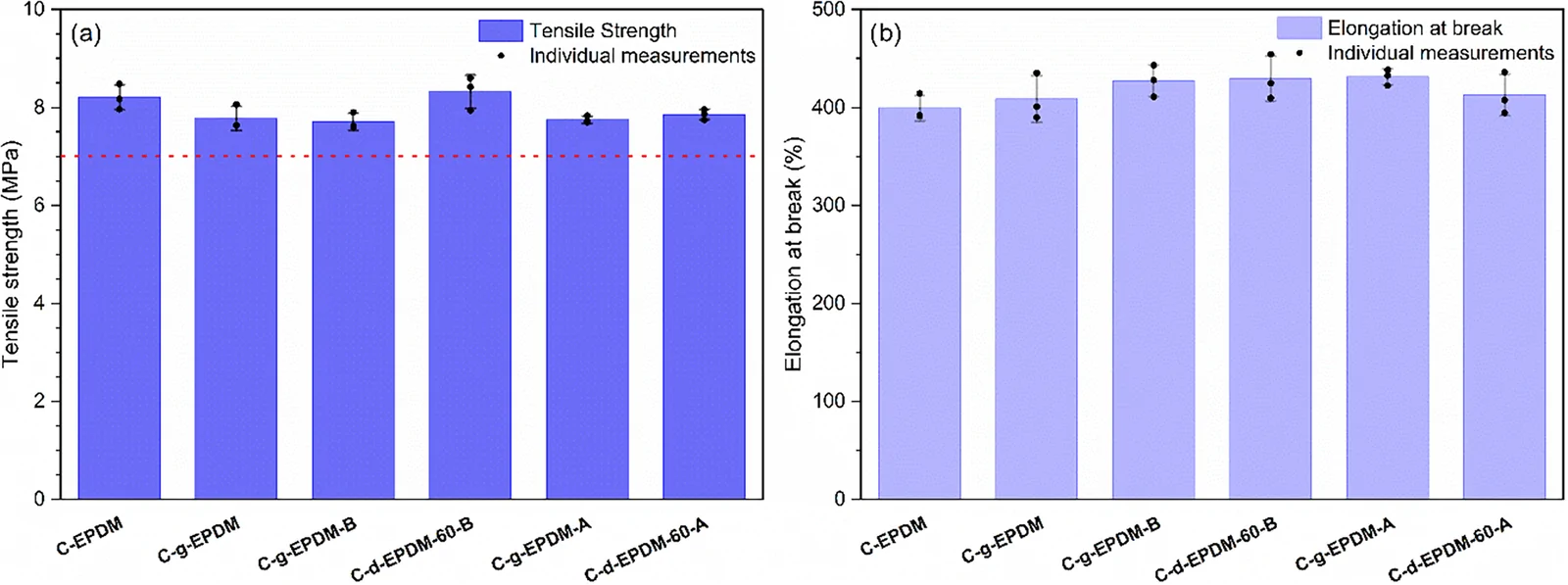
(a) Tensile strength and (b) elongation at break of the revulcanized EPDM compounds. Values are presented as mean ± standard deviation (*n* = 3). The asterisk indicates a statistically significant difference between C-g-EPDM-B and C-d-EPDM-60-B (*p < 0.05*, Tukey’s HSD test). The horizontal dashed line at 7 MPa indicates the application-related target value for automotive sealing systems in ref 67.

**Table 5:** Tensile Strength and Elongation at Break of the Revulcanized EPDM Compounds^a^

	tensile strength (MPa)	elongation at break (%)
C-EPDM	8.21 ± 0.26	396 ± 13
C-g-EPDM	7.78 ± 0.25	409 ± 22
C-g-EPDM-B	7.71 ± 0.17	423 ± 15
C-d-EPDM-60-B	8.32 ± 0.34	416 ± 25
C-g-EPDM-A	7.75 ± 0.07	428 ± 7
C-d-EPDM-60-A	7.86 ± 0.11	405 ± 27

a Values are presented as mean ± standard deviation (*n* = 3).

One-way ANOVA showed a significant difference among the six formulations (*F*(5,12) = 4.240, *p* = 0.019). Tukey’s HSD test showed that only the difference between C-g-EPDM-B and C-d-EPDM-60-B was significant (*p* = 0.046). None of the recycled compounds differed significantly from the reference C-EPDM. Two-way ANOVA showed a significant effect of microwave treatment on tensile strength (*F*(1,8) = 9.740, *p* = 0.014), while the particle-size effect was not significant (*F*(1,8) = 3.290, *p* = 0.107). The interaction approached but did not reach statistical significance (*F*(1,8) = 4.728, *p* = 0.061). The effect of microwave treatment was therefore more evident in the fine fraction, although a particle-size-dependent response could not be statistically confirmed. However, considering the narrow tensile strength range of the present formulations, the observed change should be regarded as limited rather than as a marked improvement.

Elongation at break remained within the range of 396–428% for all formulations. No significant difference was found among the six formulations (*F*(5,12) = 1.143, *p* = 0.391), and two-way ANOVA showed no significant effects of particle size, microwave treatment, or their interaction. Overall, the incorporation of untreated or microwave-treated recycled EPDM maintained the tensile properties of the reference compound. A small but statistically significant increase in tensile strength was observed only for the microwave-treated fine fraction.

## Conclusion

In this study, the effects of microwave exposure time and particle size on the response of industrial EPDM waste were investigated at a constant power of 600 W. The results show that both parameters influence the balance between network loosening and secondary cross-link formation during microwave treatment. The most pronounced network alteration was obtained for the fine fraction treated for 60 s (d-EPDM-60-B), which showed the highest apparent sol fraction (25.39%) and the lowest apparent cross-link density (0.72 × 10^–4^ mol/cm^3^), corresponding to an apparent reduction of 17.11% relative to the untreated ground EPDM. However, the swelling-derived values do not identify the chemical bonds affected and should not be interpreted as direct evidence of selective sulfur-bond cleavage. When exposure time was increased beyond 60 s, the extent of network loosening decreased. Apparent cross-link density increased relative to the corresponding 60 s-treated samples, while negative apparent devulcanization values were observed at 180 s in both fractions and from 120 s onward in the coarse fraction. This trend indicates an increasing contribution from network-forming reactions, which may include radical recombination, oxidation, and secondary cross-link formation.

XPS analysis of the 60 s-treated samples showed a decrease in the relative contribution of C–S-related carbon environments and the appearance of oxidized sulfur species at the particle surface. In contrast, ATR-FTIR showed no appreciable shifts in the principal EPDM bands. The TGA results also showed that the fine and coarse untreated fractions differed before microwave exposure, both in their degradation behavior and in the amount of residual mass. When each treated sample was compared with its corresponding untreated fraction, microwave treatment caused only moderate shifts of the main degradation peaks toward lower temperatures. Furthermore, SEM showed a similar dependence on particle-size fraction: needle- and flake-like morphological features were more evident in the fine fraction, while the treated coarse fraction displayed nodular features. Taken together, these observations show that the initial particle-size fraction played an important role in both the thermal and morphological response of the material.

In the revulcanized compounds, the unsieved formulation showed the highest mean *R*_a_ and *R*_*z*_ values. Two-way ANOVA showed no significant effects of particle size, microwave treatment, or their interaction on *R*_a_. For *R*_*z*_, the particle-size effect was close to significance (*p* = 0.052), while microwave treatment and the interaction were not significant. Shore A hardness remained approximately 75 for all formulations. Tensile strength also varied within a limited range of 7.71–8.32 MPa, and none of the recycled compounds differed significantly from C-EPDM. Within the specimen-level dataset, the microwave-treatment term was significant for tensile strength (*p* = 0.014); however, Tukey’s HSD test showed only a small significant increase between the untreated and treated fine fractions (*p* = 0.046). No significant difference was found for elongation at break. Overall, the addition of 10 phr recycled EPDM maintained the mechanical properties of the reference compound.

Overall, the findings show that the response of filled industrial EPDM waste to microwave exposure cannot be described as a simple devulcanization process. Network-loosening and network-forming reactions occurred in competition, and their relative contributions depended on both treatment time and particle-size fraction. Short exposure to the fine fraction produced the greatest apparent network loosening, whereas longer exposure times were associated with an increasing contribution from network-forming effects. These findings point to a narrow operating window in which useful network modification may be achieved without promoting excessive secondary cross-linking.

## Supplementary Material



## Notes

Factor-level coding; individual tensile-strength and elongation-at-break data; engineering stress–strain curves for all tested specimens and representative formulations; one- and two-way ANOVA results; Tukey HSD pairwise comparisons; individual surface-roughness measurements; and corresponding two-way ANOVA results for surface roughness (Tables S1–S7 and Figures S1–S2) (PDF)
