# Supplementary material for: Microwave-Treated Structural and Network Changes in Filled Industrial EPDM Waste: Effects of Particle Size and Exposure Time
Source: ACS Omega. 2026 Oct 7;11(41):62838–51. doi: 10.1021/acsomega.6c04739 (PMC13688528; doi:10.1021/acsomega.6c04739)
Supplement: Supplementary file 1 [file ao6c04739_si_001.pdf]

SUPPORTING INFORMATION

Microwave-Treated Structural and Network Changes in Filled Industrial EPDM Waste: Effects of Particle Size and Exposure Time

Begüm UZUNBAYIR [1,2], Bahadır UYULGAN [2], Orçun SAF [3]*

[1] Dokuz Eylul University, The Graduate School of Natural and Applied Sciences, Department of Metallurgical and Materials Engineering, Buca, 35390, Izmir, Türkiye
[2]Dokuz Eylul University, Department of Metallurgical and Materials Engineering, Buca, 35390, Izmir, Türkiye
[3] Standard Profil Ege, R&D Department, Manisa, Türkiye

***Corresponding Author: bahadir.uyulgan@deu.edu.tr**

This Supporting Information provides the individual mechanical-property and surface-roughness measurements, engineering stress–strain curves, factor-level coding, and detailed statistical results supporting the manuscript. Tensile data represents three specimen replicates from the same formulation batch, whereas surface-roughness data represents five spatial measurements on one plate per formulation; thus, they describe within-batch or within-plate variability rather than independent production-batch variability. Statistical analyses were performed using unrounded data at $\alpha = 0.05$, and values are reported as mean ± standard deviation.

Table S1. Factor-level coding used in the two-way ANOVA

Particle-size level	Microwave-treatment level	Formulation
**Coarse (A)**	Untreated	**C-g-EPDM-A**
**Fine (B)**	Untreated	**C-g-EPDM-B**
**Coarse (A)**	60 s	**C-d-EPDM-60-A**
**Fine (B)**	60 s	**C-d-EPDM-60-B**

Table S2. Individual tensile strength and elongation at break results for the EPDM formulations (n=3)

A. Tensile strength

	Specimen 1 (MPa)	Specimen 2 (MPa)	Specimen 3 (MPa)	Mean ± SD (MPa)
**C-EPDM**	7.969	8.484	8.180	8.21 ± 0.26
**C-g-EPDM**	7.633	8.062	7.633	7.78 ± 0.25
**C-g-EPDM-B**	7.602	7.906	7.625	7.71 ± 0.17
**C-d-EPDM-60-B**	8.602	8.422	7.945	8.32 ± 0.34
**C-g-EPDM-A**	7.703	7.719	7.836	7.75 ± 0.07
**C-d-EPDM-60-A**	7.750	7.961	7.875	7.86 ± 0.11

B. Elongation at break

	Specimen 1 (%)	Specimen 2 (%)	Specimen 3 (%)	Mean ± SD (%)
**C-EPDM**	389.0	410.9	388.3	396 ± 13
**C-g-EPDM**	431.9	407.5	388.2	409 ± 22
**C-g-EPDM-B**	424.8	436.6	407.7	423 ± 15
**C-d-EPDM-60-B**	444.4	397.7	406.3	416 ± 25
**C-g-EPDM-A**	433.3	420.8	431.2	428 ± 7
**C-d-EPDM-60-A**	406.2	376.3	431.2	405 ± 27

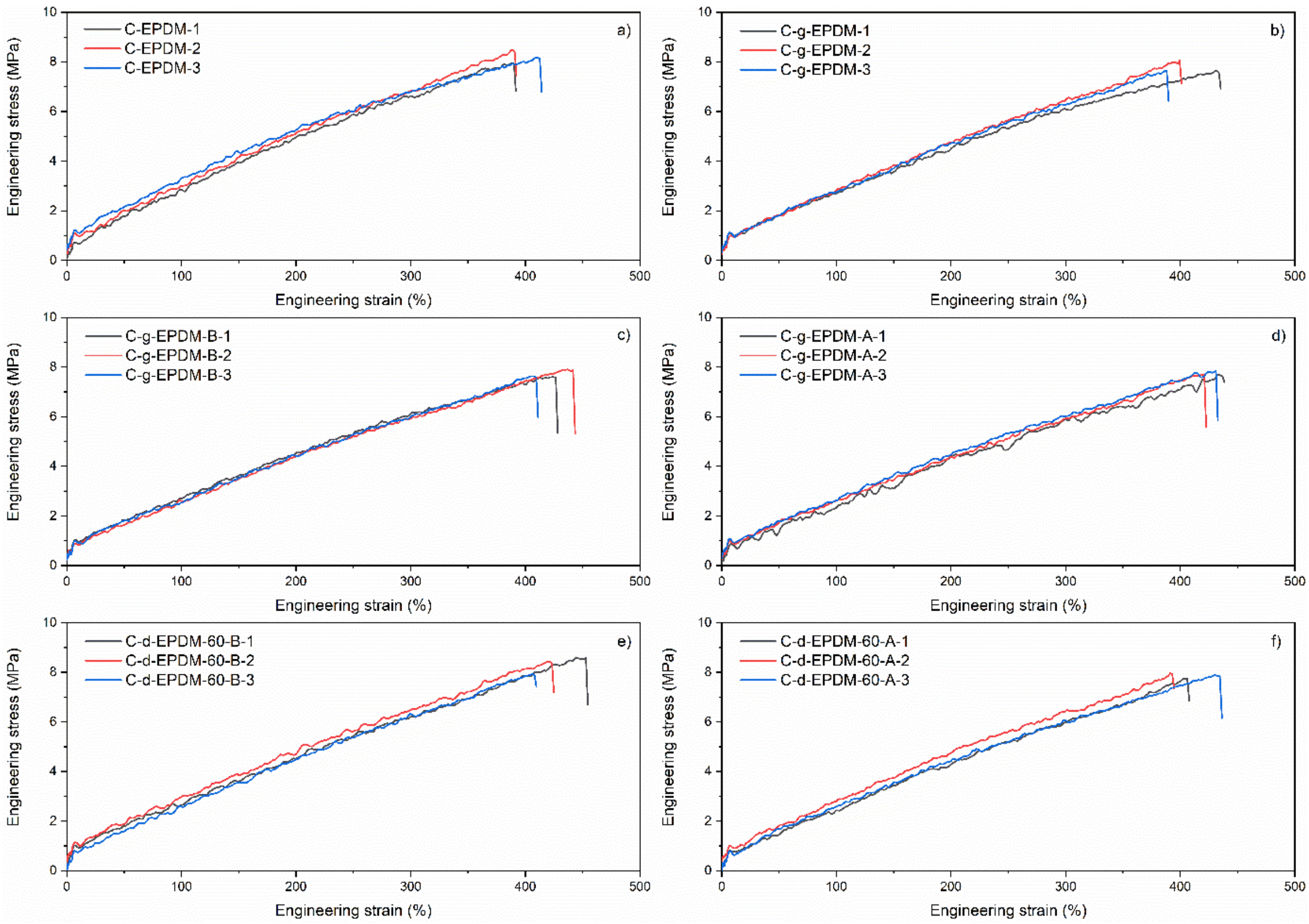

Figure S1. Engineering stress–strain curves of all three tested specimens for each revulcanized EPDM formulation: (a) C-EPDM, (b) C-g-EPDM, (c) C-g-EPDM-B, (d) C-g-EPDM-A, (e) C-d-EPDM-60-B, and (f) C-d-EPDM-60-A.

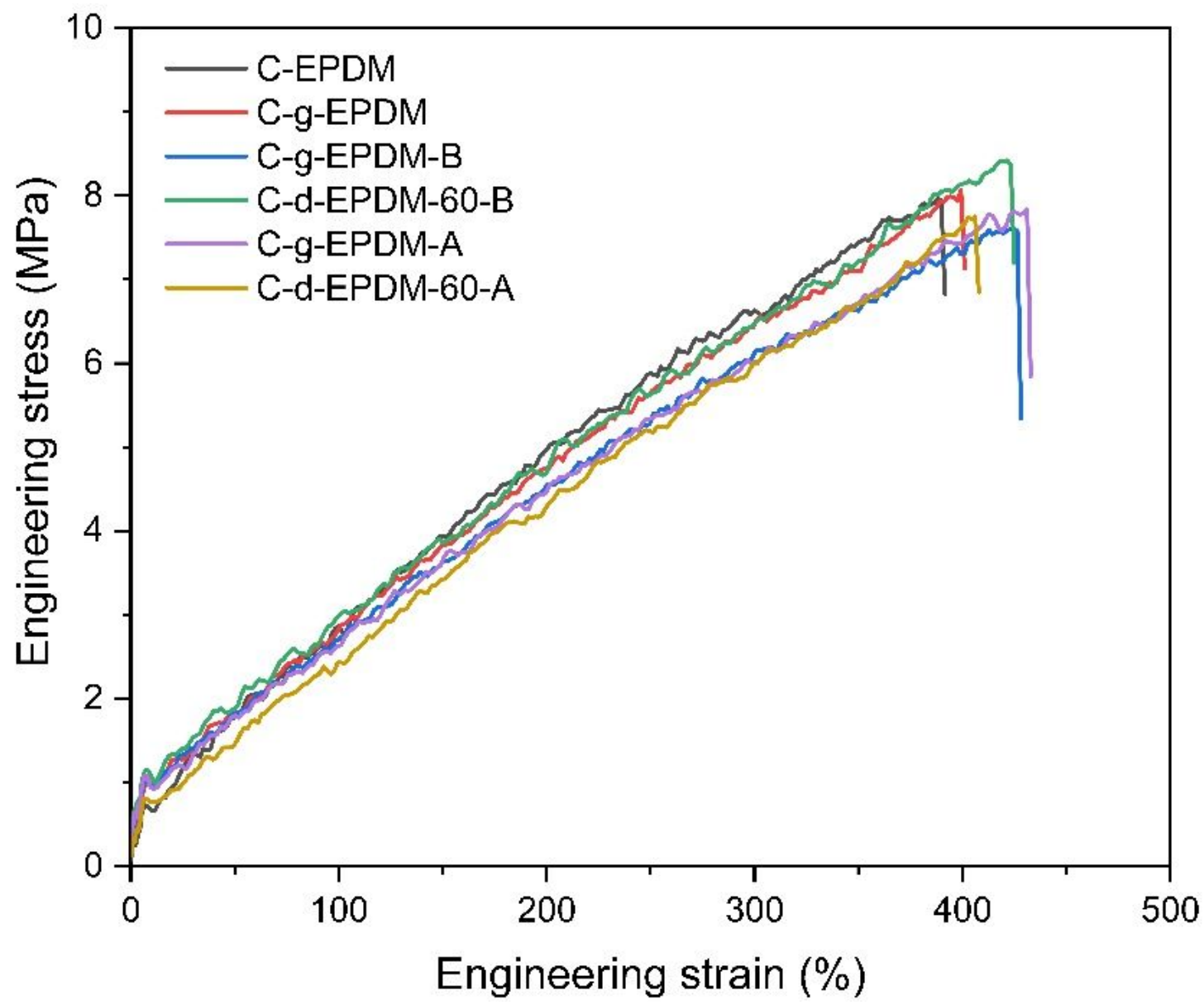

Figure S2. Representative engineering stress–strain curves of the revulcanized EPDM formulations. For each formulation, the specimen with a tensile-strength value closest to the corresponding group mean selected.

Table S3. One-way ANOVA results for tensile strength and elongation at break across all six EPDM formulations

Response	Model df	Error df	F	p	Interpretation
**Tensile strength**	5	12	4.240	0.019	Significant
**Elongation at break**	5	12	1.143	0.391	Not significant

Table S4. Tukey HSD results for tensile strength following the significant one-way ANOVA

Comparison	Mean difference (MPa)	95% CI (MPa)	Adjusted p	Result
**C-g-EPDM – C-EPDM**	−0.436	−1.038 to 0.167	0.220	Not significant
**C-g-EPDM-A – C-EPDM**	−0.458	−1.061 to 0.144	0.182	Not significant
**C-g-EPDM-A – C-g-EPDM**	−0.022	−0.625 to 0.580	1.000	Not significant
**C-d-EPDM-60-A – C-EPDM**	−0.349	−0.951 to 0.253	0.423	Not significant
**C-d-EPDM-60-A – C-g-EPDM**	0.087	−0.515 to 0.689	0.996	Not significant
**C-d-EPDM-60-A – C-g-EPDM-A**	0.109	−0.493 to 0.712	0.988	Not significant
**C-g-EPDM-B – C-EPDM**	−0.500	−1.102 to 0.102	0.127	Not significant
**C-g-EPDM-B – C-g-EPDM**	−0.064	−0.667 to 0.538	0.999	Not significant
**C-g-EPDM-B – C-g-EPDM-A**	−0.042	−0.644 to 0.561	1.000	Not significant
**C-g-EPDM-B – C-d-EPDM-60-A**	−0.151	−0.753 to 0.451	0.953	Not significant
**C-d-EPDM-60-B – C-EPDM**	0.112	−0.490 to 0.714	0.989	Not significant
**C-d-EPDM-60-B – C-g-EPDM**	0.548	−0.055 to 1.150	0.083	Not significant
**C-d-EPDM-60-B – C-g-EPDM-A**	0.570	−0.032 to 1.173	0.067	Not significant
**C-d-EPDM-60-B – C-d-EPDM-60-A**	0.461	−0.141 to 1.063	0.179	Not significant
**C-d-EPDM-60-B – C-g-EPDM-B**	0.612	0.010 to 1.214	0.046	Significant

Table S5. Two-way ANOVA for tensile properties

Response	Effect	df	F	p	Interpretation
**Tensile strength**	Particle size	1, 8	3.290	0.107	Not significant
**Tensile strength**	Microwave treatment	1, 8	9.740	0.014	Significant
**Tensile strength**	Particle size × treatment	1, 8	4.728	0.061	Not significant
**Elongation at break**	Particle size	1, 8	0.070	0.798	Not significant
**Elongation at break**	Microwave treatment	1, 8	1.743	0.223	Not significant
**Elongation at break**	Particle size × treatment	1, 8	0.537	0.485	Not significant

Table S6. Individual surface-roughness measurements for EPDM formulations (n=5 measurements on one plate)

A. R_a measurements

	Position 1 (µm)	Position 2 (µm)	Position 3 (µm)	Position 4 (µm)	Position 5 (µm)	Mean ± SD (µm)
**C-EPDM**	0.40	0.45	0.46	0.39	0.42	0.42 ± 0.03
**C-g-EPDM**	0.68	0.59	0.64	0.75	0.66	0.66 ± 0.06
**C-g-EPDM-B**	0.50	0.72	0.63	0.51	0.5	0.57 ± 0.10
**C-d-EPDM-60-B**	0.62	0.58	0.65	0.61	0.54	0.60 ± 0.04
**C-g-EPDM-A**	0.64	0.53	0.66	0.63	0.58	0.61 ± 0.05
**C-d-EPDM-60-A**	0.65	0.79	0.56	0.60	0.57	0.63 ± 0.09

B. R_z measurements

	Position 1 (µm)	Position 2 (µm)	Position 3 (µm)	Position 4 (µm)	Position 5 (µm)	Mean ± SD (µm)
**C-EPDM**	2.85	2.54	2.88	2.72	2.46	2.69 ± 0.19
**C-g-EPDM**	4.79	4.05	4.41	4.97	4.21	4.49 ± 0.39
**C-g-EPDM-B**	3.26	4.27	4.38	3.45	3.51	3.77 ± 0.51
**C-d-EPDM-60-B**	4.09	3.84	3.95	3.91	3.30	3.82 ± 0.30
**C-g-EPDM-A**	4.11	3.82	3.79	4.79	4.31	4.16 ± 0.41
**C-d-EPDM-60-A**	4.20	4.72	3.83	3.92	4.25	4.18 ± 0.35

Table S7. Two-way ANOVA for surface roughness parameters

Response	Effect	df	F	p	Interpretation
**Ra**	Particle size	1, 16	1.055	0.320	Not significant
**Ra**	Microwave treatment	1, 16	0.628	0.440	Not significant
**Ra**	Particle size × treatment	1, 16	0.001	0.977	Not significant
**Rz**	Particle size	1, 16	4.427	0.052	Not significant (near threshold)
**Rz**	Microwave treatment	1, 16	0.032	0.861	Not significant
**Rz**	Particle size × treatment	1, 16	0.004	0.948	Not significant
